# Enhancing Executive Function and Neural Health in Bipolar Disorder through Reasoning Training

**DOI:** 10.3389/fpsyg.2016.01676

**Published:** 2016-11-01

**Authors:** Erin E. Venza, Sandra B. Chapman, Sina Aslan, Jennifer E. Zientz, David L. Tyler, Jeffrey S. Spence

**Affiliations:** ^1^Center for BrainHealth, The University of Texas at DallasDallas, TX, USA; ^2^Advance MRI, LLCFrisco, TX, USA; ^3^Private PracticeDallas, TX, USA

**Keywords:** bipolar disorder, cerebral blood flow, cognition, cognitive training, executive function, frontal networks, memory

## Abstract

Cognitive deficits in executive function and memory among individuals with bipolar disorder (BD) are well-documented; however, only recently have efforts begun to address whether such cognitive deficits can be ameliorated through cognitive training. This pilot study examined the effects of a top–down, cognitive reasoning training program in adults with BD on both brain and cognitive measures. Twenty-seven participants (11 males, 16 females), aged 21–70 years old, completed the study. Participants completed neurocognitive testing and functional magnetic resonance imaging (fMRI) before and after training, consisting of 8 h (2 h/week) of training in small groups. The training delivered information processing strategies that were implemented and applicable to a variety of daily living contexts. Results indicated that participants showed significant gains in the primary outcome measure of complex abstraction, also referred to as gist reasoning, as well as in untrained domains of executive function and memory. We found a significant increase in resting cerebral blood flow (CBF) in left inferior frontal gyrus after cognitive training. We also found that resting CBF in the right frontal middle gyrus correlated positively with performance on the measure of complex abstraction. This feasibility study provides promising evidence that short-term reasoning training can enhance cognitive performance and brain health in adults with BD. These data motivate further efforts to explore adjuvant therapeutics to improve cognitive performance and underlying brain systems in bipolar, as well as other psychiatric disorders. Clinicaltrials.gov Identifier: NCT02843282, http://www.clinicaltrials.gov/ct2/show/NCT02843282

## Introduction

Bipolar disorder (BD), a mental illness with recurring episodes of mania and depression, can have far-reaching detrimental effects on the everyday function and living of those with the diagnosis. In addition to causing distressing shifts in mood and energy level, we now know that patients with BD experience cognitive deficits, not only during mood episodes but also in remission (van Gorp et al., 1998; Quraishi and Frangou, 2002; Martinez-Aran et al., 2004). A meta-analysis of cognitive deficits in adult euthymic patients with BD (i.e., neither depressed nor manic) consistently found deficits in executive function and verbal learning, with variance in deficits across other areas of cognition, such as memory, abstraction, set-shifting, sustained attention, and inhibition (Robinson and Ferrier, 2006). Robinson’s study also suggested such cognitive deficits in euthymic individuals with BD are consistent across cultures (Robinson and Ferrier, 2006).

Imaging studies also reveal underlying neural abnormalities, including changes in brain blood flow and activation. For instance, O’Connell et al. (1995) examined cerebral blood flow (CBF) in BD and identified decreased CBF in prefrontal cortex during unipolar and bipolar depression, with schizophrenic and manic bipolar individuals exhibiting even greater hypofrontality. Benabarre et al. (2005) further supported this finding of hypofrontality by correlating poorer performance in executive function and memory with low perfusion in the frontal region. Resting state functional imaging studies of individuals with BD demonstrate abnormal activation in the prefrontal and cingulate cortices (Blumberg et al., 2003; Phillips et al., 2003, 2008; Kronhaus et al., 2006; Green et al., 2007). Other studies, including a quantitative meta-analysis review of functional magnetic resonance imaging studies in BD, extend previous evidence of attenuated activation of the left inferior frontal gurus (IFG) across emotional and cognitive tasks (Phillips et al., 2008; Chen et al., 2011). Whereas cognitive and brain abnormalities in both symptomatic and asymptomatic individuals with BD are well-documented, available clinical interventions continue to be limited to pharmacological and/or psychological counseling regimens to manage symptoms. These approaches will continue to be a vital aspect of BD treatment; however, additive benefits may be derived from protocols that directly address the common cognitive sequelae.

Emerging research has examined whether individuals with BD benefit from non-pharmacological approaches to improve the cognitive symptom complex of BD, such as cognitive training, with promising results suggesting an array of potential gains in performance (Deckersbach et al., 2010; Preiss et al., 2013; Torrent et al., 2013). Previously reported studies have implemented cognitive training alone or in conjunction with other symptom management and therapeutic interventions (e.g., mood management and psychoeducation) (Deckersbach et al., 2010; Preiss et al., 2013; Torrent et al., 2013; Demant et al., 2015). The cognitive training portion of these studies targeted specific processing deficits in BD, such as memory, executive function, and attention. Studies by Deckersbach et al. (2010) and Preiss et al. (2013) resulted in significant cognitive and mood improvement after training, specifically with gains on measures of executive function and reductions in reported depressive symptoms. On the other hand, similarly well-constructed studies to address cognitive and psychosocial deficits in BD failed to find significant improvement in cognitive function (Torrent et al., 2013; Demant et al., 2015). While cognitive gains were not reported, the Torrent et al. (2013) participants did exhibit significantly improved scores on a functional outcome measure. These studies, while varied in results, support the rationale for investigating component-specific cognitive training with individuals with BD.

Based on this foundation of evidence that targeting specific cognitive processes may benefit individuals with BD, we were interested in testing whether a top–down strategy-based cognitive training of integrative processes could reap brain and cognitive gains as has been uncovered in other populations (Gamino et al., 2010; Vas et al., 2011, 2016a; Mudar et al., 2013; Cook et al., 2014; Chapman et al., 2015). We were particularly interested in measuring the benefits of gist-reasoning training, which we will refer to as ‘reasoning training,’ in this population since prior work has shown reasoning training to generalize to other cognitive domains more than targeting specific processes, albeit in different populations (Gamino et al., 2010; Vas et al., 2011, 2016a; Mudar et al., 2013; Cook et al., 2014; Chapman et al., 2015). Evidence from a series of studies from our lab has found that strategy-based reasoning training (described below) improved cognitive functions in both primary and secondary measures across several different populations including individuals with mild cognitive impairment (MCI), traumatic brain injury (TBI), as well as healthy adults and teenagers (Gamino et al., 2010; Anand et al., 2011; Vas et al., 2011, 2016a; Chapman and Mudar, 2014; Chapman et al., 2015, 2016; Mudar et al., 2016). Randomized trials comparing reasoning training to a new learning intervention in adults with TBI in chronic stages post-injury showed significant gains in abstraction, memory, executive functions of working memory and switching, non-verbal reasoning, and daily function (Vas et al., 2011). A larger randomized trial in adults with TBI found similar improvements in measures of executive function, memory and daily function (Vas et al., 2015). In this latter study, the reasoning training was shown to significantly improve psychological health with reduced depression and stress-related symptoms. A comparison of reasoning training versus a new learning intervention in adults with MCI also resulted in significant improvements in executive function and memory measures in the reasoning group (Mudar et al., 2016). A more recent study with healthy adults examined both cognitive and neural changes after reasoning training and found significant improvements in a number of frontally mediated executive functions (Chapman et al., 2015). More notably, this integrative cognitive training induced a number of brain changes at rest, including increased global and regional CBF in the default mode network and central executive network, greater connectivity in these same regions, and increased white matter integrity in the left uncinate (Chapman et al., 2015).

Previous cognitive training approaches with BD have focused largely on targeting specific cognitive processes and measuring improvement through cognitive batteries alone. Additionally, previous training protocols (e.g., cognitive remediation) have tended to require a lengthy time commitment, sometimes up to 24 weeks. In contrast, strategy-based, integrative processes can be trained over a relatively short time-span with training in ways to incorporate strategies into daily life routines. Based on principles of experience-driven plasticity, the likelihood of increased usage throughout daily life may increase chances of achieving spill-over affects into other cognitive domains, maximizing treatment efforts. For instance, in the aforementioned studies which trained reasoning, participants showed improvements in the primary outcome measure, as well as generalized cognitive benefits in other secondary outcome measures of memory and executive functioning skills (Vas et al., 2011, 2015; Chapman et al., 2015).

To date, no known study has investigated the effectiveness of a reasoning training program in individuals with BD. Additionally, of the cognitive trainings completed within the BD population, none have coupled neurocognitive and imaging measures. Therefore, the current phase I pilot trial fills a void by providing preliminary evidence of cognitive and neural benefits from a cognitive training program in individuals with BD. The current study had three major objectives. First, we proposed that a reasoning program, Strategic Memory Advanced Reasoning Training (SMART), would not only improve performance on the primary domain of complex abstraction, but would also show transfer effects to untrained domains of executive function and memory. Our second major goal was to determine whether training induced neuroplasticity changes as measured by resting CBF using pseudo-continuous arterial spin labeling (pCASL) magnetic resonance imaging (MRI). Recent studies demonstrate that CBF is reflective of neuronal health (Chen et al., 2013). Lastly, we were interested in whether the cognitive gains would be linked to specific brain blood flow changes to elucidate possible mechanisms of improvement.

## Materials and Methods

### Participants

A total of 27 adults with a diagnosis of BD I or II between the ages of 21 and 70 (see **Table 1**) participated in this study. Participants were recruited at the University of Texas Southwestern Medical Center, through psychiatrist referral, flyers and website advertising. All participants were native English speakers, had a minimum of high school education, and provided written consent in accordance with the Institutional Review Board (IRB) of our academic institutions: The University of Texas at Dallas and The University of Texas Southwestern Medical Center. Participants underwent a telephone screen with a research clinician, including a brief medical questionnaire covering their history, current medications and any pre-existing conditions. After the participant met the requirements covered by the phone screen, they were asked to complete a neurocognitive testing battery, including the Mini Mental State Exam (MMSE). The inclusion of partially (in addition to fully) remitted individuals served to confirm sufficient sample size and was based on evidence suggesting that residual affective symptoms have no major effects on objective cognitive function (Burdick et al., 2012). If subjects scored ≥26 on the MMSE and ≤17 on the Hamilton Depression Rating Scale (HAM-D), they were invited to continue with the intervention portion of the study. In addition, participants were administered a standard MRI prescreening form to assess the presence of contraindications for MRI compatibility (e.g., non-removable metal within/on the body, claustrophobia, pregnancy, non-correctable vision problems, head trauma, and CNS disease). Diagnosis of BD and euthymic state was confirmed with collaborating psychiatrist. When the participant was not a patient of the collaborating psychiatrist, written approval was obtained from the treating psychiatrist confirming the following: participant was between ages 21 and 70; participant had a diagnosis of BDI or BDII; participant was in a euthymic, rather than manic or depressive, state; participant had been stable on medications for at least 3 months; and participant was believed to be appropriate for the study. All patients were taking mood stabilizing medications and reported remaining on a consistent dose of medications throughout the study (see **Table 1**).

**Table 1:** Subject characteristics.

Participant demographic	(Mean ± SD)
**Total participants (*n*)**	**27**
Age at study entry (years)	45.8 ± 12.9
Gender (M/F)	11/16
Education (years)	16.0 ± 1.8
Age of onset (years)	34.0 ± 12.3
Duration of illness (years)	11.8 ± 9.7
Mini Mental State Examination (MMSE)	27.7 ± 1.2
Hamilton Depression Rating Scale (HAMD)	5.9 ± 3.6
**Medications (*n*)**	**27**
Lithium	8
Anticonvulsant	13
Antipsychotic	17
Antianxiety	4
Antidepressant	7
Benzodiazepines	4

### Procedure

This was a phase I, non-randomized, pilot intervention study examining the neurocognitive and brain changes from a reasoning training program, SMART, in euthymic individuals with BD. Outcome measures were administered at baseline (pre-training, T1) and within 2 weeks after completing the training sessions (post-training, T2). All 27 participants received SMART in small groups of 5–7 people. Groups were led by the same licensed speech-language pathologist. Groups met for 2 h once a week for 4 weeks (total training of 8 h). Groups were formed in order of enrollment and schedule alignment.

### Reasoning Training

Strategic Memory Advanced Reasoning Training equips participants with meta-cognitive strategies of Strategic Attention, Integrative Reasoning, and Innovation as a guide for engaging in deeper-level innovative thinking across real-life activities (Chapman and Kirkland, 2014). Strategic Attention targets the ability to filter irrelevant information in order to focus on the important information. Integrative Reasoning targets the ability to abstract meanings from specific key details (be it from a book, medical or legal advice, a movie, conversation, meeting, or other daily interaction), and to interpret within a broader context of world knowledge to create global themes and take away messages that remain relevant to the information or task at hand. Innovation targets perspective-taking, fluency, and novel idea generation in topics or areas of life that have become stagnant.

At the end of each session, participants were given homework assignments that required use of the strategies. Homework was discussed in the initial portion of the following session, and each participant reported completing the assignments. Exercises utilized a variety of materials, varying in complexity (e.g., articles, artwork, podcasts, current events, TED Talks, etc.) in multiple modalities. Emphasis was put on application of strategies to other contexts (e.g., personal relationships, work environment, and daily responsibilities). The three strategies were presented in sessions one and two, leaving sessions three and four for (1) participant demonstration of how they were incorporating the strategies in their daily lives, (2) participant-generated exercises that required implementation of the strategies (exhibiting further understanding of material), and (3) feedback, questions, and broader conversations focusing on how to practically and independently implement the strategies in daily life, as this was the final goal.

### Outcome Measures

#### Neurocognitive Measures

A battery of neurocognitive measures was administered on a non-training day at two time periods, i.e., baseline/pre-training (T1) and within 2 weeks after training (T2). Cognitive assessment measures were completed in individual, face-to-face testing sessions. All test batteries were pencil and paper measures administered by a trained research clinician (see **Table 2**).

**Table 2:** Brief description of outcome variables (scaled scores used if not otherwise specified below).

Variable	Measure	Description
**Primary outcome measure**		
Complex abstraction	Test of Strategic Learning (TOSL) (Chapman et al., 2002)	Participant synthesizes information into overview. Score: number of abstracted ideas
**Secondary outcome measures**		
Concept formation	Similarities (Wechsler, 1997a)	Participant explains what pairs of words have in common
Problem solving	D-KEFS Card Sorting Task (Delis et al., 2001)	Participant sorts six cards into two groups of three based on eight different sorting rules
Verbal fluency	Controlled Oral Word Association (COWA) (Benton et al., 1994; Spreen and Strauss, 1998)	Participant says as many words/minute as they can that begin with the given letter
Memory	Logical memory (Wechsler, 1997b)	Participant orally recalls (immediate and delayed) details of a short story read aloud
	Memory for key facts/details (TOSL) (Chapman et al., 2002)	Participant recalls (after probe) specific important facts/details from the text. Score: 0–24
	Rey Auditory Verbal Learning Test (RAVLT) (Rey, 1964; Schmidt, 1996; Spreen and Strauss, 1998)	Participants are given a list of 15 unrelated words repeated over five different trials and are asked to recall. After an interference list is given, the client must again recall the original list of 15 words and then again after 30 min. Score: 0–15 per trial
Working memory	Digits backward (Wechsler, 1997b)	Participant orally recalls number strings in backward order
Inhibition	D-KEFS Color-Word Task, Condition 3 (Delis et al., 2001)	Participant names the color of the ink a work is printed in versus reading the word (color word different than color ink)
Switching	Trails B	Participant alternately connects a set of numbers and letters in ascending alphabetical order
	D-KEFS Color-Word Task, Condition 4 (Delis et al., 2001)	Participant alternates from reading color of printed word and stating ink color of printed word. Score: total time to complete
Daily function	Quality of life in bipolar disorder (Michalak and Murray, 2010)	A 56-item self-report questionnaire on 12 domains of quality of life: Physical, Sleep, Mood, Cognition, Leisure, Social, Spirituality, Finances, Household, Self-esteem, Independence and Identity and two optional domains (Work and Education). Score: 56–280, higher score is associated with higher quality of life

The primary outcome measure of complex abstraction was evaluated using the Test of Strategic Learning (TOSL) (Chapman et al., 2002). Complex abstraction is also referred to as gist reasoning. The TOSL is an assessment that has been previously utilized as a criterion-referenced measure of ability to abstract meaning from complex information in typically developing youth (Chapman et al., 2012; Motes et al., 2014), healthy adults (Anand et al., 2011; Vas et al., 2016a), adults with MCI (Mudar et al., 2016), and adults and youth with TBI (Vas et al., 2011, 2015, 2016a; Cook et al., 2014). Recent evidence supports TOSL as both a sensitive and specific metric of complex abstraction ability (Vas et al., 2016b). Participants read a complex text (approximately 600 words). To evaluate complex abstraction, participants are then instructed to generate a high-level summary of the text. Another subtest of TOSL assesses the ability to recall key facts from the text. The TOSL complex abstraction measure has a manualized objective scoring system where each abstracted idea in the summary receives one point and verbatim or paraphrased ideas do not receive any points, the final score then reflects the total number of accurately abstracted ideas from the text. Different versions of the TOSL were administered at pre- and post- testing.

Secondary outcome measures included tests of executive function (verbal reasoning, problem solving, switching, verbal fluency), memory (including recall for details from TOSL text), complex attention, and quality of life (see **Table 2**). Recall for details from TOSL text yields a maximum possible score of 24. The Quality of Life in Bipolar Disorder (QoL.BD) was created by the Collaborative Research Team to study psychosocial issues in BD (Michalak and Murray, 2010). The QoL.BD scale is the only quality of life questionnaire specifically designed for individuals with BD. The full version has 56 questions within 10 basic domains: physical, sleep, mood, cognition, leisure, social, spirituality, finances, household, self-esteem, and two optional domains (work and education).

### MRI Experiment

Magnetic resonance imaging investigations were performed on 12 participants (six males/six females), as the rest of the participants exhibited contraindications for MRI compatibility (non-removable metal within/on the body and claustrophobia). Imaging was performed on a 3 Tesla MR system (Philips Medical System, Best, The Netherlands). A body coil was used for radiofrequency (RF) transmission and an 8-channel head coil with parallel imaging capability was used for signal reception. The MRI scans of participants were performed at rest with their eyes open and on a non-training day. We used a pCASL sequence to measure CBF at rest (Aslan et al., 2010) as well as a high resolution T1 weighted image as an anatomical reference. The details of imaging parameters and their processing techniques are provided below.

Imaging parameters for pCASL experiments were: single-shot gradient-echo EPI, field-of-view (FOV) = 240 × 240, matrix = 80 × 80, voxel size = 3 mm × 3 mm, 27 slices acquired in ascending order, slice thickness = 5 mm, no gap between slices, labeling duration = 1650 ms, time interval between consecutive slice acquisitions = 35.5 ms, TR/TE = 4020/14 ms, SENSE factor 2.5, number of controls/labels = 30 pairs, RF duration = 0.5 ms, pause between RF pulses = 0.5 ms, labeling pulse flip angle = 18°, bandwidth = 2.7 kHz, echo train length = 35, and scan duration 4.5 min. The hypercapnia BOLD imaging parameters were: single shot gradient echo EPI sequence, TR/TE/flip = 2000 ms/25 ms/80°, 43 axial slices, slice thickness = 3.5 mm, FOV = 220 mm × 220 mm, matrix size = 64 × 64 and scan duration = 7 min. The high resolution T1 weighted image parameters were: Magnetization Prepared Rapid Acquisition of Gradient Echo (MPRAGE) sequence, TR/TE = 8.3/3.8 ms, shot interval = 2100 ms, inversion time = 1100 ms, flip angle = 12°, 160 sagittal slices, voxel size = 1 mm × 1 mm × 1 mm, FOV = 256 mm × 256 mm × 160 mm, and duration 4 min.

### MRI Analysis

The pCASL MRI data underwent routine processing (Aslan et al., 2010). PCASL image series were realigned to the first volume for motion correction (SPM5’s realign function, University College London, UK). All datasets were within the applied motion threshold of 3 mm translation and 3° rotation. An in-house MATLAB (MathWorks, Natick, MA, USA) program was used to calculate the difference between averaged control and label images. The difference image was then corrected for imaging slice delay time to yield CBF-weight image, which was normalized to the brain template from Montreal Neurological Institute (MNI). Lastly, the absolute CBF was estimated in the units of mL blood/min/100 g of brain tissue (Aslan et al., 2010). The absolute whole brain blood flow values were calculated by averaging all the voxels in the absolute CBF (aCBF) map. In voxel based analyses (VBA), the individual aCBF maps were spatially smoothed [with full-width half-maximum (FWHM) of 4 mm] to account for small differences in sulci/gyri location across subjects.

### Statistical Analyses

Each neurocognitive measure was considered a dependent variable in a standard linear model. We did not omit subjects whose baseline measures were not paired with their respective post-training measures from lack of follow-up. Thus, the intent-to-treat analyses necessitated the use of a mixed model with time as a within-subject fixed factor and subjects themselves as a random factor. Temporal contrasts, which estimated mean change across the two measurement times, were of primary interest; and all hypotheses were one-sided. That is, we expected only improvements post-training. Given the inclusion of partially (in addition to fully) remitted individuals with BD, we included, in another set of models, the depression measure HAM-D as a covariate with possible interactions temporally. In these models temporal contrasts were conditional on the mean HAM-D estimate across subjects. In addition, we included age as a covariate as well as indicators for the presence or absence of concurrent antipsychotic and anticonvulsive medications to assess any potential effects on the neurocognitive measures. Finally, we applied the Benjamini-Hochberg method to control the false discovery rate (FDR) due to the large number of statistical tests on the neurocognitive battery.

For the voxel-based imaging analyses, each subject had paired measurements pre- and post-training. Thus, voxel level analyses were paired *t*-tests. Statistical inference, however, was at the cluster level using AFNI’s *3dclustsim* bootstrap method conditional on a cluster-defining threshold (*p* = 0.05) applied to all voxel-level statistics. At this threshold, contiguous clusters of voxels exceeding 1,449 (11,592 mm^3^) in number (volume) were significant at a 0.05 level, corrected for multiple clusters. We also implemented a VBA with age adjusted TOSL scores as a covariate to find potential brain regions where changes in CBF are associated with changes in complex abstraction as measured by TOSL. In this case cluster-level inference proceeded as just described, except that the cluster-defining threshold was *p* = 0.005 and the cluster size threshold for a corrected inference level of 0.01 was 263 voxels (2,104 mm^3^).

## Results

### Neurocognitive Measures

All 27 participants completed 8 h of training. **Table 3** summarizes results for primary and secondary outcomes. Most significant changes include measures of executive function and memory. Executive function measures WAIS Similarities (concept formation) (*p* = 0.004) and DKEFS Card Sorting (problem solving) (*p* = 0.001) improved significantly from pre- to post-training. Immediate memory recall improved on TOSL memory for details (*p* = 0.002). RAVLT scores improved across trials (*p* = 0.008), at a short delay (*p* < 0.001), and at long delay (*p* = 0.020). Our primary measure of complex abstraction did show improvement from pre- to post-training, although not based on the FDR criterion. This is similarly true for Trails B, Digits Backward, and Color-Word (inhibition). When HAM-D was included as a covariate, results were consistent.

**Table 3:** Summary of results for each cognitive domain (mean, standard error).

	Pre-training	Post-training	*p*-value
**Primary outcome measure**			
**Complex abstraction**			
TOSL	2.35 (0.32)	3.04 (0.32)	0.040
**Secondary outcome measures**			
**Executive function**			
WAIS, similarities	12.63 (0.40)	13.61 (0.41)	0.004^∗∗^
DKEFS card sorting: correct sorts	11.70 (0.42)	13.56 (0.42)	<0.001^∗∗^
DKEFS card sorting: description	11.74 (0.43)	12.96 (0.43)	0.001^∗∗^
DKEFS card sorting: recognition	11.56 (0.47)	12.89 (0.47)	0.007^∗∗^
Trails B	73.44 (5.43)	66.86 (5.48)	0.052
Verbal fluency	11.30 (0.61)	11.82 (0.61)	0.121
**Memory**			
Logical memory, immediate	13.85 (0.59)	13.30 (0.59)	0.812
Logical memory, delay	11.89 (0.72)	12.26 (0.72)	0.278
TOSL – memory for details	13.26 (1.00)	16.37 (0.98)	0.002^∗∗^
RAVLT, trial 5	11.96 (0.48)	12.74 (0.48)	0.008^∗∗^
RAVLT, short delay	9.74 (0.65)	11.44 (0.65)	<0.001^∗∗^
RAVLT, long delay	9.70 (0.68)	10.93 (0.68)	0.019^∗^
**Complex attention**			
Digits backward	6.78 (0.36)	7.30 (0.36)	0.039
D-KEFS color-word, condition #3	10.12 (0.54)	11.04 (0.54)	0.023
D-KEFS, color-word, condition #4	11.04 (0.37)	11.46 (0.37)	0.092
**Everyday function questionnaires**			
Quality of life in bipolar disorder	176.93 (6.90)	183.93 (7.00)	0.095

*FDR = 0.10; **FDR = 0.05.

### MRI Experiment

**FIGURE 1:**
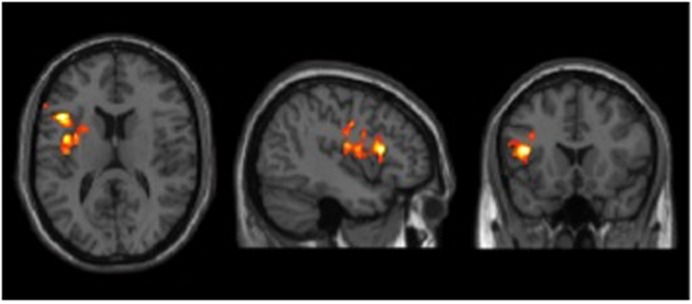
**Results of cerebral blood flow (CBF) voxel based comparison superimposed on T1 image.** Bipolar disorder (BD) participants showed a CBF increase in left inferior frontal gyrus from T1 to T2 after cognitive training, *p* < 0.05 (FWE cluster corrected) and *k* ≥ 11,592 mm^3^.

CBF was measured by pCASL MRI in BD participants’ pre- and post-cognitive training. The global CBF did not change significantly from T1 to T2; 51.2 ± 9.8 mL/100 g/min and 52.3 ± 7.8 mL/100 g/min, respectively (*p* = 0.40). The VBA was conducted on relative CBF maps, which included dividing the aCBF maps by the whole brain aCBF. In a prior investigation, we had shown that such technique improves sensitivity of regional differences by reducing physiological variations (Aslan et al., 2010). **Figure 1** shows the VBA results between T1 and T2. The BD group showed a significant increase in blood flow in left IFG after cognitive training (T1 < T2). However, no change was detected in the reverse contrast (T1 > T2). **Table 4** summarizes these findings.

**Table 4:** Cerebral blood flow (CBF) regions that showed significant blood flow change at rest in bipolar disorder (BD) group from T1 to T2.

			*MNI*			
Brain regions	BA	Cluster size (mm^3^)	*X*	*Y*	*Z*	*T*-value
*Pre-Training < Post-Training*						
Left Inferior Oper. Frontal G	13/45/6/44	11,752	-42	18	14	6.79
*Pre-Training > Post-Training*						
None						

*FWE cluster level, corrected p < 0.05, voxel threshold at p = 0.05 and K = 11,592 mm^3^.*

### Neural Correlates of Brain and Cognition

**FIGURE 2:**
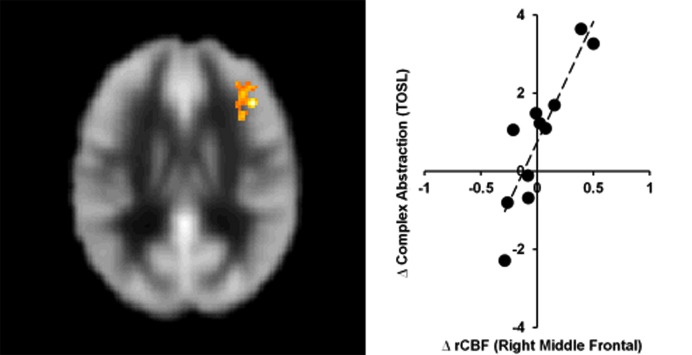
**Bipolar disorder group showed significant association between gains in regional CBF and behavioral measures.** The BD group’s TOSL complex abstraction score difference showed significant association to right middle frontal CBF increase at T2, cluster-level *p* < 0.01 (FWE corrected).

Whole brain voxel-wise correlation analysis between complex abstraction (i.e., TOSL Complex abstraction score and CBF maps, T2–T1) showed significant correlation to right middle frontal CBF; MNI coordinates: [+42 +30 +22], *t*-score of 7.6 and cluster size = 263 voxels (2,104 mm^3^, FWE cluster-level *p* < 0.01), shown in **Figure 2**.

## Discussion

This feasibility study is the first known attempt to investigate both cognitive (i.e., executive function, memory, complex attention) and brain (i.e., CBF) changes in response to a top–down, strategy-based cognitive training protocol in BD. The novel aspects of this pilot trial are testing a cognitive training protocol (SMART) and brain measurement (CBF) that have previously shown promise in studies focused on harnessing cognitive and neural plasticity. As stated above, SMART teaches top–down, metacognitive strategies that can be utilized in everyday life and its efficacy is supported by prior evidence of improved executive function and frontoparietal networks in other clinical and healthy populations (Chapman et al., 2015, 2016; Vas et al., 2015; Mudar et al., 2016). Previous neurobehavioral interventions in BD have focused largely on improving the psychological health with only more recent focus on targeting the cognitive deficits that have now been well-documented (Deckersbach et al., 2010; Preiss et al., 2013; Torrent et al., 2013; Demant et al., 2015). Additionally, recent technological advancements in resting state MRI allow measurement of neuroplasticity changes with CBF, which is beginning to show promise as one potential objective neural marker of enhanced brain health. This potential measurement of neuroplasticity change is derived from a well-accepted coupling between healthy neural activity and cerebrovascular flow (Raichle and Gusnard, 2002; Chapman et al., 2016), as well as accumulating evidence that different forms of interventions show increased CBF with concomitant improvement in cognitive functions (Takeuchi et al., 2013; Chapman and Mudar, 2014; Chapman et al., 2015, 2016; Vas et al., 2016a).

Three major findings emerged from this pilot study. First, we found that a strategy-based reasoning training, delivered over 4 weeks with 2 h each week for a total of 8 h, improved complex abstraction abilities in adults with BD. Moreover, the training showed generalized benefits to other cognitive domains that were not specifically trained, including specific executive functions (i.e., concept abstraction and problem solving) and memory (word lists at immediate and delayed periods and details from complex information). Secondly, we found significant pre to post increases in CBF in the left prefrontal cortex, namely in the left inferior frontal gyrus (LIFG), a region of the brain associated with semantic and cognitive control processes (Poldrack et al., 1999; Gold et al., 2006; Badre and Wagner, 2007; Danker et al., 2008; Race et al., 2009). Third, we found a positive correlation between enhanced cognition (complex abstraction) and increased resting CBF in the right prefrontal cortex, supporting the potential for the training to positively impact both brain and cognition; in other words, enhance brain health.

Our findings of improved executive functions in BD with SMART concur with recent evidence from other trainings that impairments in executive function in BD can be mitigated with cognitive training (Deckersbach et al., 2010; Preiss et al., 2013). The trainings in these two prior studies trained a broad-base of specific processes whereas our training taught strategies to deal with complex information and contexts encountered in everyday life. Additionally, we found significant improvement on memory measures, a cognitive domain which has not previously shown to be enhanced in other cognitive training studies with BD. These findings support SMART training as an integrative, top–down process that promotes not only synthesizing and abstraction of information, but can also potentially promote deeper encoding and recall of information. Current results add to prior evidence that cognitive training of higher-order integrative functions, which equip individuals with strategies to employ throughout their daily routine after training has ended, could be a beneficial intervention, when used to complement standard of care therapeutic approaches in BD.

Since both our training and the Preiss et al. (2013) study took only 8 h of training, we propose that short-term cognitive interventions may be worth the cost in terms of time and money since they appear to provide benefits in mitigating cognitive deficits in BD. Moreover, the present results add to the emerging evidence that neurotherapeutic management and overall life functionality in BD may be improved when the cognitive deficits are addressed along with the psychiatric symptoms of depression (Deckersbach et al., 2010). The current results suggest the degree of depressive symptoms was not a contributing factor that impacted response to cognitive training, a promising finding which suggests these psychiatric problems are not necessarily a limiting factor to treatment benefit. Treatment of psychiatric disorders, e.g., BD, and other brain disorders has far too long been siloed with sole focus on only one domain of brain health rather than taking into account other aspects of brain health, both cognitive and psychological health. For example, the psychiatric symptoms in BD tend to be the sole focus of management without addressing the co-occurring cognitive deficits. In a contrasting focus, in acquired brain injury the key focus has been on remediating executive functions without concern for the co-morbid psychiatric problems, such as depression and anxiety which are being identified (Didehbani et al., 2013; Max et al., 2015). The potential for cognitive training to show spillover effects to improving psychological health benefits is intriguing. In a previous study, we found that SMART training in adults with TBI showed not only cognitive gains but also psychological benefits that were manifested by a significant reduction of depressive and stress-related symptoms that continued to improve 3 months after the training ceased (Vas et al., 2016a). Unfortunately, we failed to measure post-treatment psychological health in BD in the present study. Nonetheless, Deckersbach et al. (2010) showed that cognitive training was associated with a decrease in residual depressive symptoms as well-increased occupational and overall psychosocial functioning immediately and at 3 month follow up.

In addition to improvement on neurocognitive measures, imaging results showed increased regional brain blood flow in the left prefrontal cortex. There is considerable controversy as to whether increased CBF is associated with improved brain function (Sojkova et al., 2008) or compensatory reaction. Much of the controversy arises in activation studies, but less so in resting state CBF studies. For example, activation studies have measured both increases and decreases in BOLD signal during mental activation tasks. What is important to note, however, is that this change is a transient effect and is typically restored to baseline level when the brain returns to resting state (Raichle and Gusnard, 2002). Therefore, as a tightly regulated system, resting CBF is remarkably consistent and an important biomarker (Raichle and Gusnard, 2002). Additionally, results from a growing number of studies reveal that resting state CBF may represent a promising neural marker of brain response to distinct interventions across clinical populations (Mozolic et al., 2010; Vas et al., 2015). These studies identify a correlation between increased CBF and improved cognitive function with healthy agers and those with brain injury (Takeuchi et al., 2013; Chapman et al., 2015, 2016; Vas et al., 2015). We propose that the increased resting CBF in the present study of individuals with BD reflects a similar improvement. Specifically within the frontal lobe, we found a significant increase in resting CBF in the LIFG (which showed a significant gain from pre- to post-training). Other researchers have supported the pivotal role of the LIFG as being associated with cognitive control processes, such as executive function (Poldrack et al., 1999; Gold et al., 2006; Badre and Wagner, 2007; Danker et al., 2008; Race et al., 2009). What is intriguing and perhaps strengthens the present results, is that we also previously reported increased resting CBF in the LIFG at post SMART training in other populations (Chapman et al., 2015; Vas et al., 2015).

Thirdly, we found a significant association between increased CBF in the right middle frontal gyrus (MFG) and improved complex abstraction. We speculate that the reasoning training engaged cognitive control to facilitate processing of complex information that also involved fine-tuning of attentional systems to inhibit less important information with concomitant improvement in prefrontal cortex (Chapman et al., 2016). Supporting this plausible explanation, Corbetta et al. (2008) have shown the right MFG to be a potential modulator between attention networks. Additionally, the right frontal cortex has previously been associated with increased CBF being linked to higher complex abstraction performance (Chiu Wong et al., 2006).

Whereas the current results require further examination in larger clinical trials, the capacity to induce increased resting CBF in the prefrontal cortex of individuals with BD as a result of integrative cognitive training could have beneficial clinical implications. This is the first known study to investigate brain changes in response to cognitive training in BD with findings suggesting improvement to brain areas that might be compromised by the disease, such as the prefrontal cortex (Blumberg et al., 2003; Phillips et al., 2003, 2008; Kronhaus et al., 2006; Green et al., 2007). In sum, we propose that the increased health of the frontal regions, as manifested by elevated CBF, could be a result of experience-driven neural plasticity where the concerted cognitive effort recruited and improved the health of this neural system.

### Limitations and Future Directions

This study has a number of strengths, which include utilization of brain and neurocognitive outcome measures, as well as implementation of a novel cognitive training intervention for individuals with BD. We also note a number of limitations which make us interpret our findings cautiously. The major limitations include the lack of either a waitlist or active control group and the fact that the same clinician trained and tested participants. With regard to the first limitation of no control group, we cannot rule out the possibility of practice effects; however, we do not feel that practice effects account for the gains across measures, especially given the convergence of gains in cognition and increased CBF. Additionally, in prior studies testing efficacy of SMART, which included active control or wait-list control groups, we found similar executive function and neural benefits from the reasoning training as compared to the control groups. Specifically, studies with traumatic brain injury populations demonstrated similar gains in areas of complex abstraction, executive function, and memory after training; whereas the control group exhibited no significant gains in any cognitive domains (Vas et al., 2011; Cook et al., 2014). Studies examining SMART training with healthy individuals, both adolescent and adult, resulted in significantly improved ability in measures of executive function including complex abstraction and memory, while the control group showed no significant gains in any cognitive domains (Gamino et al., 2010; Chapman et al., 2015). Similarly, in a population at-risk for Alzheimer’s, Mudar et al. (2013) found significant improvements following the SMART protocol on measures of abstraction, executive function, and memory relative to the active control group, which showed no such significant gains except for the D-KEFS sorting test. Regarding the second potential limitation of clinician bias, two clinicians, who were unaware of time of testing (i.e., pre or post) scored the key measures independently to minimize experimenter bias. Moreover, participants’ pre- to post-responses were not paired for scoring so that no comparison between pre and post response could bias change scores. We failed to find significant differences between scores of the two raters. Alternate versions of primary and secondary measures were used, when available, at pre- and post-testing to also help reduce practice effect. We recognize that future studies should include a control group as well as blind the examiners to group membership. Nonetheless, we do not feel that these two factors fully explain the current findings and believe the current results offer a promising pattern of findings that motivate a more in-depth trial.

Another potential weakness, but also strength, is the limited range of depressive symptoms. As our participants had relatively low depression scores, it would be interesting to consider whether patients with greater levels of depressive symptoms would show different degrees of benefit, perhaps more or perhaps less. The fact that the degree of affective symptoms did not affect results leaves the question open as to whether those with more serious levels of symptomatology might still benefit. A recent review concluded that cognitive effects of lithium, anticonvulsants, and antipsychotics on cognition in BD are still unknown, primarily due to flawed research methodology, small sample sizes, and inconsistent findings (Vreeker et al., 2015). In this current study, however, we did assess the potential effects of anticonvulsants and antipsychotics. Neither medication type nor its interaction with time were significantly different for any behavioral measure. With a larger sample size, we could also more clearly examine effects of medications, such as anti-psychotics and mood stabilizers, as well as dosage effects. We find the strength of the current results encouraging for conducting a larger trial to confirm these findings and consider other questions such as treatment earlier in the disease course, perhaps after the first episode of depression. Additional weaknesses are the relatively small sample size, a lack of post-testing in psychological health, testing at extended periods post-treatment to evaluate whether the gains persisted and the failure to consider occupational functionality.

The cognitive gains from the current study replicate previous findings of training-specific gains, as well as generalized improvement in cognitive skills in various clinical populations that underwent reasoning training (Anand et al., 2011; Vas et al., 2011, 2015; Mudar et al., 2016). We propose that these preliminary data motivate a larger, randomized study. A larger sample size would also allow better assessment as to what, if any, distinct effect depressive symptoms had regarding training benefits. Future investigations should also include indicators of level of engagement and long-term follow-up assessments (e.g., 6 and 12 months post) to evaluate maintenance of gains, as well as earlier in the disease course.

## Conclusion

The impact of cognitive training protocols is a particularly important line of research in BD. The potential to strengthen mental capabilities, and executive functions, in particular, may be a tangible path to motivate a productive life style course in populations with psychiatric disorders where the cognitive sequela has been relatively unexplored. Strengthening neural circuits through cognitive training in individuals with psychiatric disease may provide one promising adjuvant to the pharma-interventions to supplement individuals’ capacity with psychiatric disease to achieve higher levels of success in personal and occupational goals, as has been suggested by Fisher et al. (2009).

## Author Contribution

All authors listed, have made substantial, direct and intellectual contribution to the work, and approved it for publication.

## Conflict of Interest Statement

The authors declare that the research was conducted in the absence of any commercial or financial relationships that could be construed as a potential conflict of interest.
